# FlexiChain 3.0: Distributed Ledger Technology-Based Intelligent Transportation for Vehicular Digital Asset Exchange in Smart Cities

**DOI:** 10.3390/s23084114

**Published:** 2023-04-19

**Authors:** Ahmad Alkhodair, Saraju P. Mohanty, Elias Kougianos

**Affiliations:** 1 Department of Computer Science and Engineering, University of North Texas, Denton, TX 76203, USA; ahmadalkhodair@my.unt.edu (A.A.); saraju.mohanty@unt.edu (S.P.M.); 2 Department of Electrical Engineering, University of North Texas, Denton, TX 76203, USA

**Keywords:** blockchain, Distributed Ledger Technology (DLT), Cyber-Physical Systems (CPS), intelligent transportation (ITS), vehicle-to-vehicle communication (V2V)

## Abstract

Due to the enormous amounts of data being generated between users, Intelligent Transportation Systems (ITS) are complex Cyber-Physical Systems that necessitate a reliable and safe infrastructure. Internet of Vehicles (IoV) is the term that describes the interconnection for every single node, device, sensor, and actuator that are Internet enabled, whether attached or unattached to vehicles. A single smart vehicle will generate a huge amount of data. Concurrently, it needs an instant response to avoid accidents since vehicles are fast-moving objects. In this work, we explore Distributed Ledger Technology (DLT) and collect data about consensus algorithms and their applicability to be used in the IoV as the backbone of ITS. Multiple distributed ledger networks are currently in operation. Some are used in finance or supply chains, and others are used for general decentralized applications. Despite the secure and decentralized nature of the blockchain, each of these networks has trade-offs and compromises. Based on the analysis of consensus algorithms, a conclusion has been made to design one that fits the requirements of ITS-IOV. FlexiChain 3.0 is proposed in this work to serve as a Layer0 network for different stakeholders in the IoV. A time analysis has been conducted and shows a capacity of 2.3 transactions per second, which is an acceptable speed to be used in IoV. Moreover, a security analysis was conducted as well and shows high security and high independence of the node number in terms of security level per the number of participants.

## 1. Introduction

Every day, the number of vehicles on the road is increasing, which causes traffic congestion and delays in the transit process for emergency vehicles, such as ambulances, fire trucks and police cars [1]. Transportation solutions that were formerly acceptable have become insufficient in addressing the enormous growth in the number of vehicles over the last two decades despite significant improvements in infrastructure [2]. ITS integration is more important than ever. ITS is meant to aid in the construction of “smart roads” by decreasing the incidence of traffic jams and increasing the effectiveness of relieving them. Insight into traffic conditions and availability is provided to users. As a result, travel is safer and more pleasant, and less time is spent traveling to and from daily destinations. The IoV is a novel concept that evolved from the idea of Vehicular Ad hoc Networks (VANETs) as a result of recent advances in computing and communication technology [3,4]. For ITS to function, the IoV must first be established. The United States Department of Transportation (DOT) [5] claims that the IoV can help reduce accidents involving sober drivers. Cars can communicate with each other in order to track other cars’ movements and whereabouts [6]. The term IoV is used to describe a system in which vehicles are linked together and can share and receive information from one another and from other devices. This paves the way for the instantaneous dissemination of data regarding traffic conditions, road hazards, and other elements that might significantly affect travelers’ safety and productivity.

With the IoV in place, it is predicted that 79% of such collisions can be prevented through improved coordination and dialogue between vehicles [2]. Bicycles, pedestrians, and roadside infrastructures are all linked together, reducing environmental pollution, accident rates, and traffic jams [7,8,9] by exchanging messages about traffic conditions and information on safety and accidents with a worldwide traffic control system that improves convenience, comfort, and safety. Thus, improvements in public transportation and pedestrian traffic are also possible. Because of Global Positioning Systems (GPS) technology, it is now possible to know where other vehicles are in situations such as blind spots, stoppages on the highway but concealed from view, around a blind corner, or blocked by other vehicles. A vehicle’s ability to anticipate and respond to changing driving conditions can provide immediate warning to its owners [10]. When it comes to preventing car accidents, the primary purpose of Vehicle-to-Vehicle (V2V) communication technology is to help drivers be aware of their surroundings and increase safety at a reasonable cost.

Traditional means of interoperability in the IoV have included cellular networks, satellite communications, and Dedicated Short-Range Communication (DSRC). While these approaches have shown promise, they are not without drawbacks, including security risks and transmission inefficiencies. For example, a smart vehicle is one that can sense its surroundings and operate independently without human intervention [11]. A smart-featured automobile relies on sensors and actuators, complicated algorithms, machine learning systems, and powerful processors. Sensors in various components of the vehicle are used to construct and maintain a map of the vehicle’s surroundings. Radar sensors keep an eye out for other vehicles that may be approaching from behind. Traffic lights, road signs, other vehicles, and pedestrians are all detected by video cameras. To determine distances, road boundaries, and lane markers, Lidar sensors bounce light pulses off the car’s surroundings [12]. When parking, ultrasonic sensors in the wheels pick up on obstacles such as curbs and other vehicles. The car’s actuators, which control the car’s acceleration, braking, and steering, receive orders from sophisticated software, which interprets the sensory data and maps a route. Predictive modeling and object identification assist the software with navigating traffic regulations and avoiding obstructions [13]. All these data are generated from one smart entity, which will create a challenge. Challenges exist to scalability such as scalability in data, scalability in throughput, scalability in power, and scalability in time response.

Smart vehicles are a trending research area for many companies, labs, and researchers due to their anticipated benefits to the quality of life [14]. This type of driving relies partially on machines and is ruled by algorithms and embedded standards and regulation codes which give drivers more tools to enhance their experience. Security and real-time operations are an important factor in such applications, where the impact of any failure will influence lives. Each vehicle will be full of sensors to read the environment and act accordingly. The integration of multiple technologies will burden the central authorities regarding security threats [15]. Depending on the above, several questions come to mind, such as: How to create secure communication? How to avoid latency? How to reduce centralization? How to reduce power consumption? How to encourage nodes to act honestly? Such a large, complex CPS has many obstacles to overcome for full deployment such as interference conditions, traffic regulations, and complex V2V communications.

The introduction of the blockchain DLTs, which have altered numerous aspects of our lives, has been one of the most revolutionary developments of the past few decades [16,17]. These innovative methods of data storage and transaction processing have the potential to affect a broad range of industries, including banking, supply chain management, government, healthcare, and ITS [18].

DLT is a group of mechanisms and protocols governed by the consensus mechanism of participants through direct communication in an untrusted environment [19]. DLT has been proposed to resolve many issues of the current centralized paradigm of intelligent transportation and to provide a secure environment for its operations [20,21]. ITS-VANETs need to acquire the characteristics of DLT such as decentralization, immutability, transparency, security, efficiency, and programmability in order to satisfy its requirements as a Complex Cyber-Physical System (CCPS) [22]. Other examples include real-time interaction, scalable architecture, automated operation, low power consumption and security. Increased trust, transparency, and security are just some of the ways in which DLT could change the face of ITS [23]. Certain conditions must be met before DLT-based ITS operations can be put into place. Already existing ledger structures and consensus algorithms need trade-offs in which some times security and privacy are strong but scalability is weak or scalability is high but security is low.

Scalability: The volume of data related to transportation operations is expected to expand, and the system must be able to process a high volume of transactions and nodes. This is not the case in all DLTs. Some lack scalability such as Bitcoin. The paradigm is a fit for electronic cash systems but will cause an issue in term of scalability and hardware requirements if applied to ITS. Blockchain-Based Secure Data Exchange (BDESF) ITS is a secure and tamper-proof framework for data exchange and storage. It also prevents replay, Man-in-the-Middle (MiTM) weaknesses, impersonation, data leakage, and unwanted data updating with authentication and privacy measures. BDESF-ITS integrates smoothly with existing transportation systems. BDESF-ITS is a strong security mechanism for DLT applications to transportation security and privacy [24]. However, Practical Byzantine Fault Tolerance (PBFT) is suggested to be used in such a framework which will burden the network with the redundancy. Power consuming protocols are part of the scalability problem and need a pre-existing level of trust to be initiated. In addition, this protocol has a lower degree of decentralization which will change the nature of ITS-VANETs. Another example is interoperability: when it comes to transportation, the DLT system should be compatible with a wide range of systems, technologies, and platforms to ensure that data are shared effectively among all parties involved. With the absence of Layer 0 in the crypto-networks and due to the importance of interoperability to an ITS system, the choice of a certain ledger should be based on the requirement of ITS.

Security and Privacy: Data integrity and confidentiality must always be maintained by the system to prevent any unwanted changes or disclosures to private information. Strong encryption, access restriction, and other privacy-protecting measures fall under this category. For example, Ethereum provides strong cryptography and a medium latency which is acceptable. However, with the growth of VANETs, the network will encounter some throughput and routing issues due to time adjustment (used in Bitcoin) and the huge amount of operations which take place in Ethereum to reach agreement. In [25], a Blockchain-based Conditional Privacy-Preserving Authentication (BCPPA) protocol employs key derivation and blockchain technology to enhance VANET authentication and privacy. BCPPA utilizes Ethereum smart contracts to secure vehicle communication over VANET. The costly Elliptic Curve Digital Signature Algorithm (ECDSA) can be replaced with a modified version or another Public Key Infrastructure (PKI)-based signature with bulk verification to increase efficiency. Using blockchain technology, the BCPPA protocol provides conditional privacy-preserving authentication and decentralized, tamper-proof VANET communication. Even though smart contracts are hosted in a secure blockchain, limiting the process of securing the communication to a programmable transaction will centralize the process in addition to the centralization level of Ethereum.

Latency: Traffic management, navigation, and V2V communication are just a few examples of real-time ITS applications that require low latency. Transactions and data exchange on the DLT system must be rapid. The nature of ITS-VANETs is direct and rapid communication. When using a DLT-based framework, the latency must be taken into account. In [26], the primary contribution of this work is to propose a secure 5G-ITS through the use of blockchain technology to evaluate trust against potential attacks. To accomplish hierarchical trust evaluations and protect the privacy of users, federated deep learning is used to evaluate the trust of ITS users and task distributers. In order to guarantee the efficacy and accuracy of trust evaluation, hierarchical incentive mechanisms are also designed to implement reasonable and fair rewards and punishments. Hyperledger is used to implement frameworks and is well known for its power consuming and high resource usage. The nature of DLT-based IoV is decentralization while ensuring fairness and decentralization. In [27], a Public, Special, and Supreme framework is presented. The “Public” blockchain server is responsible for service-related data transmission, verification, and storage. It is a shared blockchain server with limited storage capacity. Once the public blockchain’s memory is complete, it will replace its own data within the blockchain. Similarly to the public blockchain server, the “Special” blockchain server displays dynamic features. Specifically, the “Supreme” blockchain server is used to store all network-participating vehicle information. Each data transmission detail of the intelligent vehicle is securely preserved and processed in the supreme blockchain. The nature of the required operation for DLT usage is not satisfied, since the three servers reduce the decentralization and increase the vulnerability toward Single-Point Failure (SPF).

Consensus Mechanism: Security, decentralization, and performance are all factors that should be considered while deciding on a consensus process. It also needs to be secure against attacks such as Sybil and 51% of attacks as well as energy efficient. Consensus is the core of any DLT. Most of the challenges faced are based on the consensus algorithm. In [22], a blockchain-enabled vehicular crowd-sensing technology secures 5G Internet of Vehicles user privacy and data safety by securing real-time traffic data. A deep reinforcement learning (DRL) algorithm selects the best active miners and transactions to optimize blockchain security and latency. A two-sided matching-based approach allocates non-orthogonal multiple access sub-channels to reduce uploading delay for all users. This technology safeguards vehicular crowd-sensing data collecting and user privacy. The consensus algorithm proposed in this work is PBFT, which is known for its high tolerance and security but has overhead computational requirements. Reference [28] proposes the Ethereum-based VNB (VANETs with a Blockchain). The VNB simulates a vehicle on-board unit (OBU), scanning adjacent vehicles, authenticating them, and communicating with blockchain accounts. The VNB correctly distinguished different vehicle types in simulations. Despite its limitations, the proposed VNB offers a promising security and trust architecture for autonomous vehicular networks and ITS in smart cities in the near future. Proof of Work (PoW) and Proof of Stake (PoS) are both used as consensus algorithms. PoW is utilized for its ease and security in determining the correct nonce, while PoS is utilized for its energy efficiency and decentralization prevention. Nonetheless, PoS is susceptible to double-spend attacks. PoW is known for its high security but needs resource-rich nodes, and thus, it is not suitable for IoV operations. PoS is known for its security and operations efficiency, but it is vulnerable to centralization and routing problems.

Other conditions and criteria such as data quality: Information saved and transmitted through the system must be as accurate and trustworthy as possible by excluding any potentially misleading data.

Governance: The many participants in the DLT-based ITS ecosystem need a well-defined governance framework that specifies their specific responsibilities and how they will make decisions.

Legal and Regulatory Compliance: The system must follow all data protection, privacy, and cybersecurity legislation, both domestically and internationally.

Incentive Mechanisms: Suitable incentive mechanisms, such as token-based rewards for users and service providers, should be built into the DLT-based ITS to encourage widespread adoption and active involvement.

User Experience: The system needs to be simple and straightforward so that those who really utilize the DLT-based ITS services can get about with ease. By meeting these requirements, a DLT-based ITS can contribute to the development of a more productive, secure, and transparent transportation ecosystem, which will benefit users, operators, and regulators. In this paper, we compare multiple blockchain and non-blockchain consensus algorithms and their applicability to serve ITS applications based on the requirement [29]. We propose FlexiChain 3.0 Technology as a platform to host ITS digital assets collections and exchange in V2V, Vehicle-to-Machine (V2M), and Vehicle-to-Human (V2H) transactions. In addition, a detailed security analysis for certain types of attacks between the proposed work and related works is presented.

**Figure 1:**
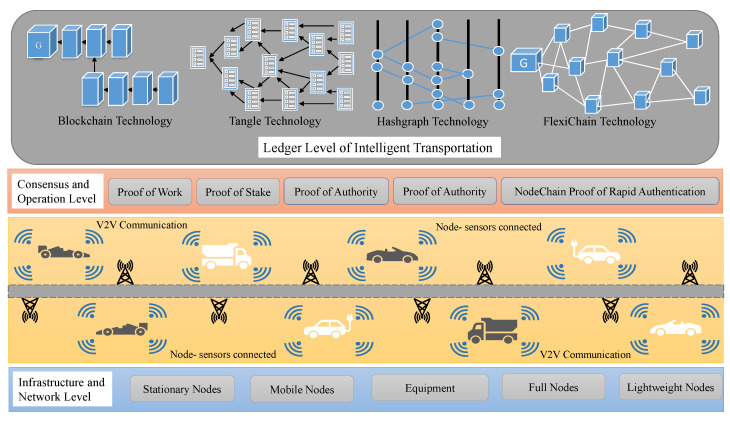
High-Level Depiction of DLT-Based ITS.

Figure 1 illustrates the layered structure of employing DLT in intelligent transportation in applications such as auto vehicle driving data training, V2X communication, vehicles’ history, and autonomous vehicles.

The rest of the paper is organized as follows: Section 2 summarizes the novel contributions of this paper. Section 3 presents background information and previous related works. Section 4 presents the proposed system. Section 5 provides experimental results. Finally, Section 6 concludes the paper and presents directions for future research.

## 2. Novel Contributions

In this section, the paper’s unique contributions are discussed, and the proposed work is highlighted. Data accuracy, instant responses, security, consistency, fault tolerance and privacy are all required for such an ITS-V2X system. The accuracy of any ITS relies on the huge data accumulation and training through an Artificial Intelligence (AI) agent which requires correct information and integrity to produce a useful feedback and directions. Security and privacy are required to keep the operations running smoothly with no fails or undesired events to keep peoples’ and nodes’ identities secured and private. Consistency is required to ensure that the operations are always on and will not encounter any issues even during an attack such as Distributed Denial of Service (DDoS). Low latency is a need, since all operations in ITS require the lowest time to execute. In addition, power consumption is a critical factor which should be minimized for sustainable and reliable operations.

### 2.1. Problem Addressed

With the advancement of technology, vehicles act as a driving computer system recording routes, status, identities, and data, and they also give feedback to users. Due to this huge amount of data generated from vehicles, a secure platform is desirable. In addition, secure channels and fast communication are also required. Due to the amount of data involved, this can be a challenge. Moreover, data training, data exchange, central authority and speed all are challenges to the current paradigm. DLTs are suitable to resolve ITS challenges but must satisfy the desirable requirements for the application. For example, using the blockchain (Bitcoin) paradigm will not benefit ITS due to its operation that by design has been targeting an electronic cash system. As another example, the blockchain (Ethereum) paradigm is a very efficient distributed super computer but the operation is suitable to web applications and financial applications where a few seconds of latency will not harm, whereas one second of latency might cause huge safety and security issues in ITS.

### 2.2. Solutions Proposed

An exploration of the feasibility for DLT-based CPS, such as ITS, is justified since the technology provides a secure platform that could make it practical and effective. An analysis of prior technologies and their consensus algorithms is presented to analyze the need of having a customized or application-based designed DLT to satisfy ITS conditions. FlexiChain 3.0 is an upgraded version from our previous work [30] for ITS data collection and trade-based ITS proposed to introduce the feasibility of operating a V2V network over FlexiChain technology [17,20,30].

### 2.3. Significance of the Solution

1. The suitability of several technologies to ITS is analyzed.
2. The need of an application-based DLT is introduced.
3. Propose a DLT that could satisfy ITS requirements without trade-offs.
4. A novel technology framework, FlexiChain 3.0, is presented as a solution.
5. The novel DLT is designed specifically for ITS as CCPS.

In Table 1, a comparison is given between our previous versions of FlexiChain and the current work. In [30], the work is representing FlexiChain 1.0, which is combining our work with the novel MultiChain Proof of Rapid Authentication [17]. A novel block structure has been proposed with an enrollment process that creates the Accessible Secure Identification List proposed in [20]. In the next version, FlexiChain 2.0, an upgrade was presented on how to generate the file using a combined ledger of NodeChain, and how the file is created and updated, making NodeChain as Layer0 and all other blockchains as Layer1. Moving to the next version, since the proposed work is about a designed distributed ledger for Cyber-Physical Systems, FlexiChain 2.0 has been upgraded and modified to fit ITS applications as a complex CPS using a distributed offline vault (NodeChain), which is a manufacturer’s predefined trust and which provides a public permissioned ledger. The rest of the table is covering other differences and similarities.

## 3. Background and Previous Related Work

### 3.1. Smart Cities

Smart cities have recently generated a lot of attention. The idea of having a smart city comprising smart sectors such as smart population, smart administration, smart transportation, smart agriculture, smart grid, smart education, and smart infrastructure is unique but challenging [32]. Due to the attention given to the notion of a smart city, it has been defined in various ways in [33]. Others have presented the current concept of smart city and its future directions [34]. In [35], an extensive overview of smart cities is presented covering a wide range of topics including research aims, research challenges, potential scenarios, and potential project areas [14]. Functional specifications that needed to be known about smart cities have been discussed in [36]. Since the quality of life for each citizen is the most important factor, designing and planning the smart services within a smart city must be completed in a way that could facilitate people’s lives due to the adoption of information and communication technology (ICT) [37]. In [38], numerous potential and commercial benefits associated with the smart economy and the connection between the economy and citizens are explored. In [39], it is stated that a smart government in smart cities is effective only if it offers their residents city services, channels, smart mobile services, and network integration. Environment quality such as air quality, water, trees, waste management, and infrastructure has been discussed in [40,41,42]. While using all these smart services, enormous amount of data are produced and have to be managed. Big data and the paradigm of the Internet of Things (IoT) or CPS might raise some challenges such as security, and privacy issues. In [43], protecting user confidentiality in blockchain-based IoT systems is discussed. The paper focuses on privacy issues presenting examples and cases. In [13], the potential of adopting DLT in smart cities and their applications is investigated. In addition, some blockchain paradigms that could be applied to a smart city are discussed.

#### NEOM

Recently, a new smart city has been introduced to the whole word under the name of “The Line” as part of the huge project of the government of Saudi Arabia’s Neom mega city. The city is built over the desert from the ground up as a smart city taking into account all aspects to provide the optimum quality of life. The city will include a smart infrastructure as well as smart supply chain and logistic services. The city is designed with smart energy systems [44,45].

### 3.2. IoV and VANET

IoV is a concept that extends the IoT to the transportation domain, allowing for seamless connectivity and communication between vehicles, infrastructure, and other smart devices [46]. IoV seeks to develop intelligent transportation systems with enhanced safety, traffic efficiency, and vehicle experience. VANETs serve an essential role in the execution of the IoV vision. The IoV incorporates a wider array of applications, devices, and technologies, whereas VANETs are primarily concerned with V2V and vehicle-to-infrastructure (V2I) communication. The contributions of VANETs to the IoV ecosystem are as follows: Communication: VANETs serve as the communication infrastructure for IoV vehicles. They enable V2V and V2I communication, facilitating real-time data sharing and decision making. Safety: By enabling vehicles to share information about their position, speed, and orientation, VANETs enables various safety applications, such as collision avoidance and early warning systems, which are integral components of the IoV [47]. VANETs enable vehicles to share traffic information such as congestion levels, road conditions, and detours, which can optimize traffic flow, reduce travel time, and enhance overall transportation efficiency in the context of IoV. Data collection and analysis: VANETs can facilitate the accumulation of vast quantities of data from vehicles and infrastructure that can be used for real-time monitoring, predictive analytics, and decision making within the IoV ecosystem. VANETs can be incorporated with other IoT systems, such as smart grids and smart cities, allowing for a more comprehensive and interconnected approach to transportation management and urban planning. VANETs are a crucial component of the IoV, as they provide the communication infrastructure required for vehicles and the infrastructure to exchange data and cooperate. VANETs contribute to the development of safer, more efficient, and environmentally friendly transportation systems within the IoV ecosystem as a whole by facilitating efficient communication between vehicles and roadside infrastructure.

### 3.3. DLT

DLT is a broad term that encompasses a variety of technologies that facilitate the secure, transparent, and decentralized storage of records across a network of participants [48]. A top–down approach to elucidating DLT would entail deconstructing the concept into its fundamental elements and then building upon them. Here is an in-depth explanation of DLT.

#### 3.3.1. Cryptography

Several cryptographic techniques and mechanisms are used in DLTs to ensure security, privacy, and data integrity. Some of the key techniques and mechanisms include:

#### 3.3.2. Hash Functions

These are mathematical algorithms that take an input and produce a fixed-size output, which is called a hash. In DLT, hash functions are used for data integrity, tamper resistance, and generating unique identifiers. Examples of hash functions used in DLT include SHA-256 (used in Bitcoin) and Keccak-256 (used in Ethereum).

#### 3.3.3. Digital Signatures

Digital signatures enable the sender of a message to sign it with their private key, proving authenticity and integrity. In DLT, digital signatures are used for transaction authorization and ownership verification. Commonly used digital signature algorithms in DLT include ECDSA and the Edwards-curve Digital Signature Algorithm (EdDSA).

#### 3.3.4. Public Key Cryptography (PKC)

Known as asymmetric cryptography, PKC uses a pair of keys, namely a public key and a private key. In DLT, public keys serve as user addresses, while private keys authorize transactions and asset transfers. Examples of PKC used in DLT are the RSA algorithm and Elliptic Curve Cryptography (ECC).

#### 3.3.5. Cryptographic Key Derivation Functions (KDFs)

KDFs are used to generate cryptographic keys from user-provided inputs, such as passwords or passphrases. In DLT, KDFs increase the security of keys, making it harder for attackers to guess or brute-force them. Examples of KDFs used in DLT include scrypt, bcrypt, and Argon2.

#### 3.3.6. Cryptographic Consensus Mechanisms

Cryptography plays a role in consensus mechanisms that maintain the integrity and security of the distributed ledger. Examples include PoW, where miners solve cryptographic puzzles to validate transactions and create new blocks, and PoS, where validators are chosen based on their stake in the network.

#### 3.3.7. Privacy-Enhancing Techniques

Cryptographic techniques are also used to preserve privacy in DLT. Some examples include:

1. Zero-knowledge proofs (ZKPs): ZKPs enable users to establish the authenticity of a statement without disclosing additional details. Examples of ZKP systems used in DLT are zk-SNARKs (used in Zcash) and zk-STARKs.
2. Confidential transactions: These techniques hide transaction amounts or other sensitive data. Examples include Pedersen commitments and Bulletproofs (used in Monero and Mimblewimble-based protocols).
3. Ring signatures: Ring signatures obscure the sender’s identity in a transaction by making it indistinguishable from other users in the same group. An example of a ring signature implementation in DLT is CryptoNote (used in Monero).

These cryptographic techniques and mechanisms form the foundation of security, privacy, and data integrity in DLT systems. As the technology evolves, new cryptographic techniques may be developed and adopted to address emerging challenges and enhance the capabilities of DLT.

#### 3.3.8. Transaction

**Figure 2:**
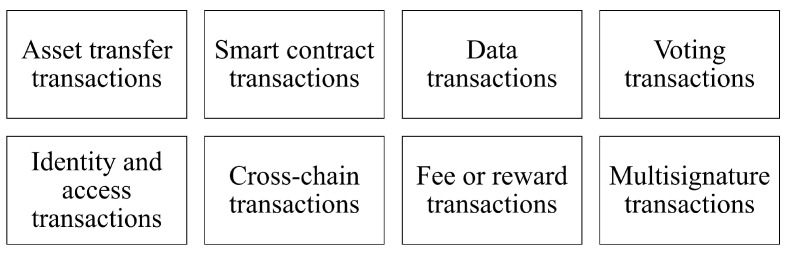
Transaction Types.

In DLT, a transaction is an operation or event that involves the transfer or modification of assets, data, or other digital resources within the network. Transactions are the fundamental building blocks of DLT systems, and they are used to record and track the history of assets and interactions among network participants, as shown in Figure 2.

#### 3.3.9. Ledger Structure

**Figure 3:**
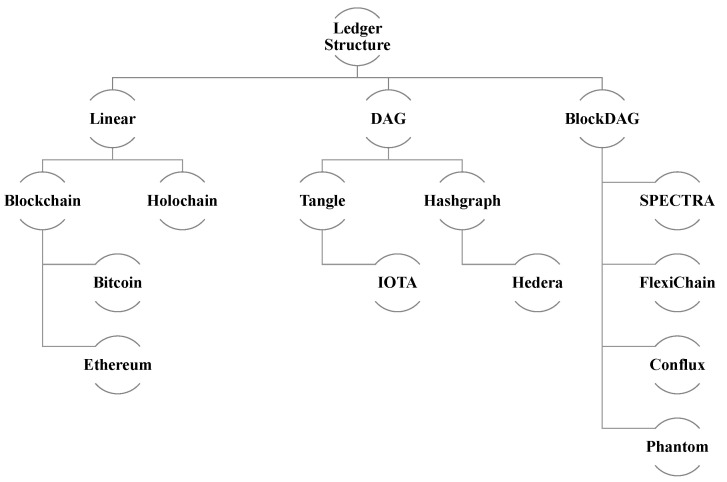
Ledger Structure.

A DLT’s ledger is a data structure that captures transactions and maintains a verifiable record of all network activity, as shown in Figure 3. The ledger structure can take various forms, such as a linear blockchain (e.g., Bitcoin, Ethereum) or a Directed Acyclic Graph (DAG), depending on the specific DLT implementation (e.g., IOTA).

#### 3.3.10. Consensus Algorithms

DLT systems utilize consensus algorithms that enable network members to agree on the validity of transactions for the purpose of maintaining a consistent and secure ledger. PoW, PoS, and Byzantine Fault Tolerance (BFT) are prevalent consensus mechanisms. Each mechanism has trade-offs regarding security, efficiency, and resource usage. The consensus algorithm is the core of the technology, and it is what supports the decentralization nature, increases the security and transparency, and is a key player in network throughput and latency. Table 2 lists some consensus algorithms and their trade-offs.

#### 3.3.11. Nodes and Network Architecture

In a DLT, the ledger is maintained by a network of nodes, or machines, that validate transactions, store the ledger, and communicate with each other. Nodes can have various duties and responsibilities, such as full nodes (which store the entire ledger) and lightweight nodes (which do not store the entire ledger but storing only a subset of the ledger). The architecture of the network is distributed and decentralized with no singular point of failure or control.

#### 3.3.12. Applications and Use Cases

DLT can be applied to a wide range of industries and use cases, such as finance (cryptocurrencies, remittance, tokenization of assets), supply chain management (provenance tracking, inventory control), healthcare (secure data sharing, patient records), identity management (digital identity, access control), and more.

### 3.4. DLT-Based ITS Related Works

Vehicles become smarter everyday due to the advancement of transportation and communication technology [56]. The blockchain can potentially handle various IoV applications with creative solutions. Most IoV applications are real time, mobile, and generate and share large amounts of data. In IoV environments, many standard strategies may not work. In addition, increased connectivity may give malevolent actors new attack channels. Blockchain incorporation into the IoV enhances security, privacy, trust, system performance, and automation. Thus, blockchain-like robust technology should be used for flexibility and big data. We list some important incentives for IoV blockchain adoption below. To be considered a “smart city”, a metropolis must have reliable public transportation. Due to the many factors that must be considered to ensure passenger safety, ITS is classified as a Complex Cyber-Physical System (CCPS). The term “Internet of Vehicles” refers to the network of vehicles that allows them to share data, conduct analysis, and deliver and receive feedback in a real-time environment [3,4].

The IoV has the potential to become the next decade’s trend due to advancements in satellite communications, AI and CPS [56,57]. The next decade will see a rise in vehicle automation and intelligence. A small number of initiatives, such as ERTICO ITS Europe and City Verve Manchester, have been launched to aid the development of ITS in smart cities. Depending on the precision of the data and the methods used to regulate traffic, a mountain of information will be generated as the number of vehicles on the road continues to swell. If the operations are conducted using the traditional IoT model, then latency, complexity, and IoV needs will be major obstacles. Compatibility and interoperability across IoV components from different service providers is extremely difficult to guarantee. Data interchange and storage infrastructure must be decentralized, distributed, inter-operable, flexible, and scalable to support the growth of IoV and unlock the full potential of ITS. Data security and management, data resource and training, resource sharing, vehicle management, ride sharing, content streaming, traffic control and management, and V2V communication are all areas where DLT could serve as a platform [18].

In [58], a blockchain-based distributed ledger solution for managing data securely within the vehicle edge computing networks using a consortium blockchain was developed. There are two ways in which the use of smart contracts would improve the proposed system. As a first step, the smart contract is utilized to guarantee the integrity of data exchanged between vehicles and the edge computing servers located in vehicles. Second, the smart contract prevents unauthorized disclosure of the data. Hence, vehicles may pick the greatest and most trusted source of high-quality data. The suggested system also includes a mechanism to share data based on its reputation. Taking into account the number of encounters, the timing of occurrences, and the closeness of their trajectories, a three-weight subjective logic model is utilized to manage the reputation of vehicles in a fair and realistic manner. The proposed system can more easily detect vehicles that are maliciously intent on harming others or acting suspiciously with the use of this reputation scheme than with more conventional reputation schemes.

In [59], with the support of a smart contract, physically unclonable functions (PUFs), and a public-key infrastructure, DrivMan was proposed, which is a blockchain-based solution for automobiles that facilitates trust management, data provenance, and privacy via PKI. DrivMan’s use of the blockchain allows for distributed trust management even in a partially trusted network. In addition, thanks to the PUFs’ role in creating a crypto fingerprint for each vehicle, DrivMan can demonstrate the origin of the data. PKI is also used to enable car registration and provide key pairs to automobiles via a Certificate Authority (CA). If necessary, the CA can track down the source of malicious vehicles’ certificates and revoke them. The purpose of PKI is to prevent attackers from discovering genuine identities and to safeguard personal information by making it impossible to connect identities to public keys.

The fundamental innovation proposed in [60] is a new method of key negotiation that allows for auditable and verifiable cryptographic signatures. In particular, this strategy aims to address concerns regarding the safety, credibility, and oversight of jointly held information. The approach allows either script-based or static automated key exchange. Because of this, crucial vehicle-to-vehicle communication talks may be completed rapidly and mechanically. In addition, the negotiation process prevents a packet-dropping attack from taking place because of its use of preventative measures such as scripts, channels, and scheduling.

Data trading proposed in [61] presents a number of challenges that can be mitigated with the use of the blockchain, including a lack of transparency and traceability as well as the potential for illegal alterations to data. To audit and verify transactions, a group of regional aggregators collaborates like a consortium. To further ensure that societal utility is maximized, data is sold at the optimal price, and individual privacy is safeguarded for both buyers and sellers, an iterative double auction method is employed. More people will share information as a result of this. The system is made more reliable by also factoring in the price of transferring data. Resource sharing between cars is another application of this technology.

The solutions proposed in [62] are ensuring that bids are transparent and are allowing buyers and sellers to engage in resource trading. A broker is proposed here as a means of maintaining the market for trade. The broker then determines the quantity of resources being traded and devises a price rule to induce truthful bidding from buyers and sellers. An iterative two-sided auction is the process proposed here. Therefore, it is the most fiscally responsible, sensible, and socially beneficial option.

The Device-to-Device Edge Computing and Networks (D2D-ECN) in [63] utilizes blockchain technology, smart contracts, edge computing, and device-to-device connectivity to facilitate asset trade and the distribution of work. When completed, D2D-ECN will serve as a shared environment for developing high-performance and low-latency applications together. This ensures that real-time application scenarios can be handled fast by offloading the computing responsibilities. Swarm intelligence is used to devise a method of work allocation that minimizes both delay and processing time. While addressing the problem of poorly managed resources, it also helps to establish trust between those who supply resource services and those who need them to complete their work. For devices with fewer resources, the proof-of-work consensus technique is replaced with proof-of-reputation. With this setup, only the user with the greatest reputation value can pack resource transactions. The blockchain serves as the repository for the reputation values. Each entity’s reputation value is calculated by factoring in both its recent and past achievements. The players are rewarded by a game-theory-based process.

The study in [64] details a blockchain-based system that safeguards the anonymity of automobile drivers when they look for and reserve parking spots in advance. The usage of blockchain technology is proposed in this proposal as a means of avoiding the drawbacks of centralization. The blockchain cannot function without the cooperation of parking lot owners even though they may not trust one another. They may include information on parking deals, for instance. Then, the blockchain will serve as a permanent record of all open offers. The private information retrieval (PIR) approach is utilized alongside the blockchain to conceal drivers’ whereabouts. When using PIR, drivers can look for parking information in the blockchain without disclosing their intended destination. After receiving a parking offer through the blockchain network, the driver can confirm the reservation with the parking owner in a way that protects their anonymity by using a short, randomizable signature. However, the trusted authority will be able to identify the genuine drivers and take appropriate action, if necessary, because of this signature’s short length and randomizability. It is also proposed that drivers have the option of making payments anonymously rather than using conventional card payment systems, which could potentially expose their personal information. A comparative view of these works is presented in Table 3.

## 4. Proposed FlexiChain 3.0: DLT-Based V2V

DLT has been proposed as a solution to multiple challenges in various applications. In this section, the DLT will be proposed as a solution to fulfill the requirements of ITS-IoV. DLT has secured operations due to its architecture and distributed form since it relies on the nodes and not a central authority. In addition, securing assets and eliminating malicious behavior is proved through some established distributed ledger networks that have been operating for several years. Nodes are independent in distributed networks, but different methods are used to track the updated state of a ledger or a digital asset. The ledger is distributed, which means each node has its own copy which reduces security threats if this technology used in ITS.

In this paper, it is assumed that trusted manufacturers are producing Trusted Modules which in this case will contain vehicle keys. These entry keys are linked to each other and are contained in a NodeChain-Assisted Distributed Offline Vault that unifies and secures the vehicles’ identities [20]. Surface Zones are represented in this work as a blockchain for each zone, and all blockchains are strongly linked through FlexiChain, which represents Layer0 for all blockchains.

FlexiChain 3.0 Technology could provide a solid ledger for ITS due to its multiple features that have been designed to target this type of application using multiple blockchains as multiple areas that cars drive through. Each car can operate in every zone due to the flexibility of Layer0 which provides to the network one-time registration. Nodes in this application represent cars, stations, towers, trucks, etc.

FlexiChain 3.0 is a Layer0 ledger that uses BlockDAG structure to build its ledger. It uses Proof of Rapid Authentication (PoRa) as its consensus algorithm that relies on trusted module authentication and lightweight computation. FlexiChain 3.0 uses NodeChain for its authentication process from which the network security independence increases. NodeChain is an integrated ledger initiated with the network and used to mirror nodes and secure their manufacturers’ specification and an agreement reached among stakeholders to add a device to create its correspondent Trusted ID (TUID) which is used in the operations of Layer0 that is represented here as zones.

### 4.1. FlexiChain 3.0 Layer0 (Zones)

**Figure 4:**
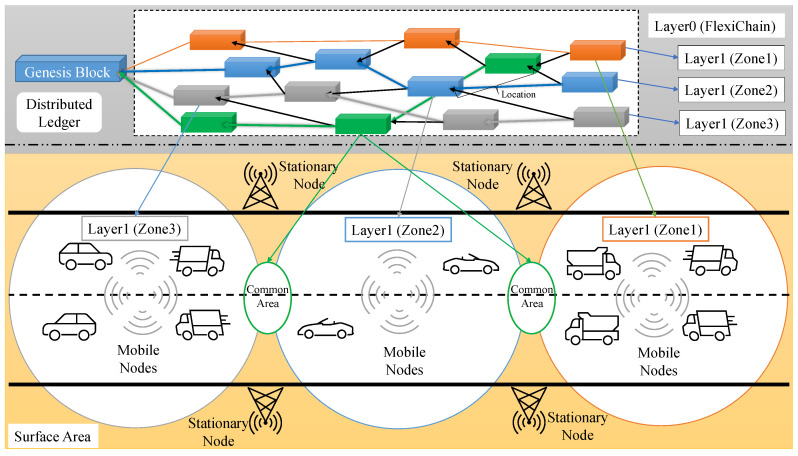
Zones and Their Corresponding FlexiChain Ledger.

In this section, the zones component of the FlexiChain-based proposed framework of Peer-to-Peer (P2P) communication system is explained and illustrated in Figure 4. In this framework, the area of the proposed application is divided into zones each of which has its own blockchain and is connected with Layer0 FlexiChain. Each blockchain has a block type and is defined to all vehicles entry keys (trusted modules).

### 4.2. Block Types (Digital Assets Collected and Exchanged)

**Figure 5:**
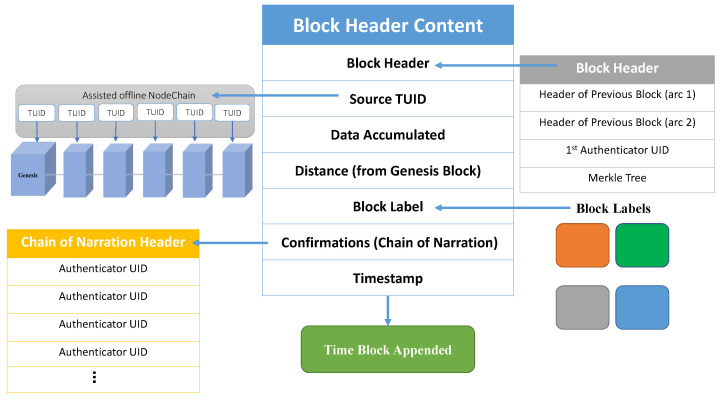
Block Content of Each Zone and its Labels.

This section shows the contents of each block. All zones have the same block structure, but they are labeled differently to append to the location specified. The block contains the header which is the hash value of all comprised data. The source TUID is included to be authenticated. The data are collected from the car or its environment. The distance from the genesis block is based on the location chosen. Minimal distance is used to obtain the shortest way to genesis if block reduction is needed. Lastly, the chain of narration is used to list all nodes that have confirmed this block to present block authenticity within the FlexiChain. Block content and types are presented in Figure 5.

### 4.3. Node Types (V2V Participant Authority Levels)

There are three types of nodes:

1. The Backup Node (BN) is the network’s “cloud”, or original node. In NodeChain [20], the first block represents the virtual existence of a backup node.
2. Vehicles or fixed stations are classified as edge nodes, and they are full nodes and have a full ledger.
3. CPS and IoT nodes or subscriber nodes are used for data collection and transmission. Since this technology is aiming for restricted nodes, a node that is both IoT and CPS could qualify based on the requirements. These represents sensors and actuators in the proposed framework.

### 4.4. Trusted Modules (Vehicles Entry Keys)

Trusted modules in this proposed framework are the vehicles’ entry keys. The keys are manufactured with a built-in signature generator and a copy of the NodeChain which gives each car access to the NodeChain-Assisted Distributed Offline Vault for rapid authentication. The initial registration process runs through the manufacturers as the stakeholders of the network. The modules provide an extra level of security to compensate the low computation required to append a block. Once this key is inserted to the car or identifies the signal of the car, the data collected by the car sensors and actuators are collected and broadcast to the ledger.

### 4.5. NodeChain-Assisted Distributed Offline Vault (Vehicles Digital Unique Identity Aggregator)

**Figure 6:**
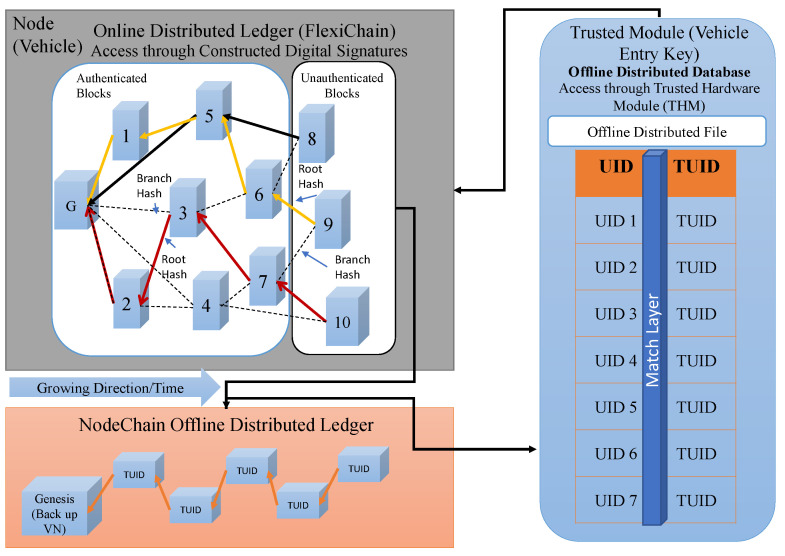
NodeChain Offline Vault.

**Figure 7:**
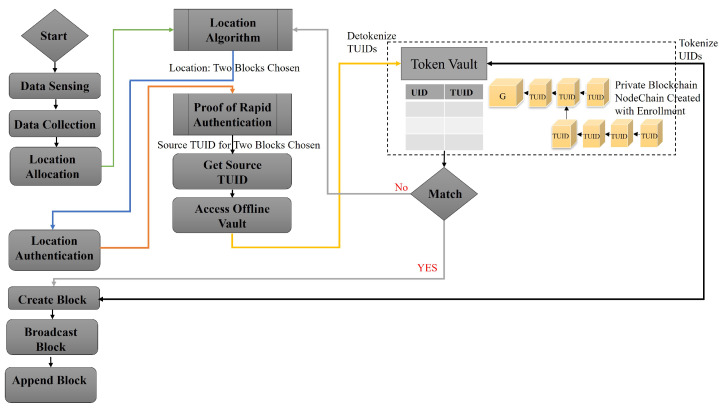
NodeChain Proof of Rapid Authentication.

NodeChain [20] is formed and built by the registration process. It has all nodes’ TUIDs, and these TUIDs are a tokenized version of the real UID that is assigned by the manufacturer and registered in the NodeChain, as shown in Figure 6. Only the vehicle’s entry keys can be accessed, and with its own signature, the real UID can be matched (Figure 7).

## 5. Experimental Results

### 5.1. Time Analysis

#### Setup

A total of 64 nodes of each technology have been created and run for 30 min, as detailed in Table 4. Docker containers have been used to host each node and pair with the second node.

**Figure 8:**
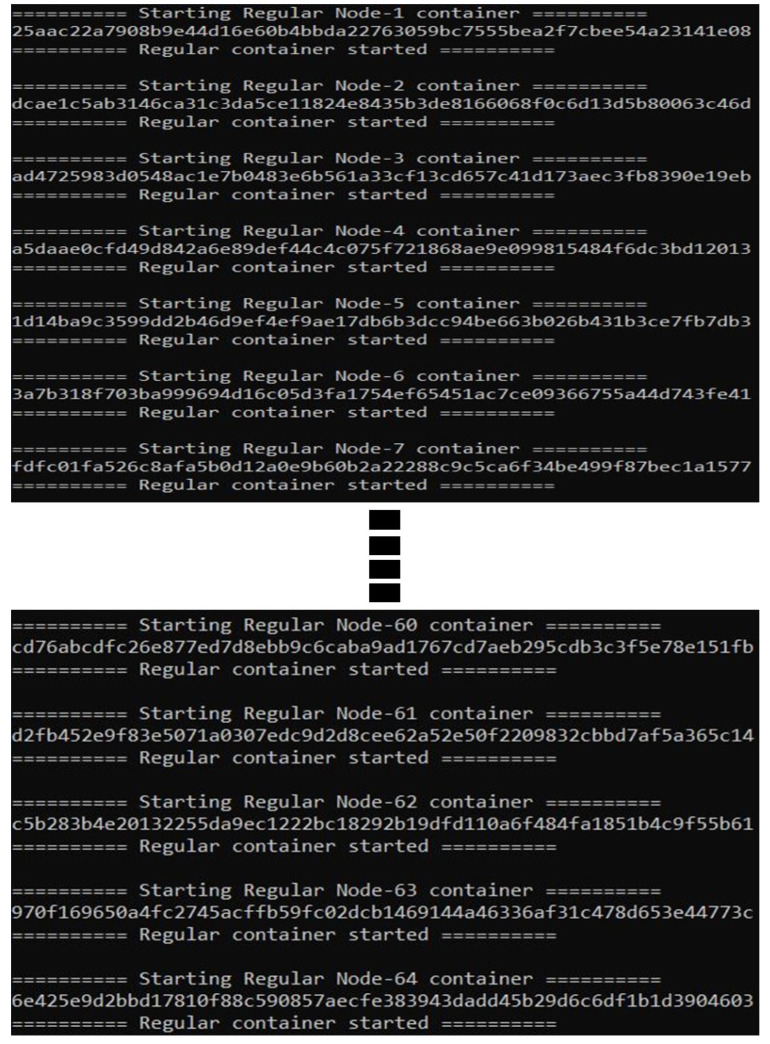
The 64 FlexiChain Nodes Registered.

**Figure 9:**
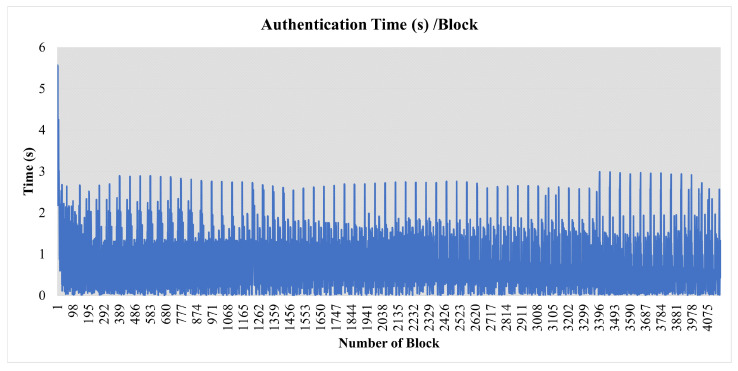
Nodes Containers on Docker.

Nodes directly send blocks to each other. FlexiChain has been implemented using Python and PostgreSQL and is running through docker containers. The network starts by running the BN. The regular nodes join after NodeChain has initialized. The initialization sequence of the network is shown in Figure 8. A performance analysis is shown in Table 5 and a real-time graph of authentication activity is shown in Figure 9.

### 5.2. Security and Privacy Analysis

FlexiChain Technology is built with CPS applications in mind; therefore, security measures are built in at both the hardware and software levels. Researchers may assess the efficacy of the technique by simulating a variety of security threats [16]. Such attacks include corrupting the exchanged data between nodes, implanting incorrect data during communication, and full malicious control over the network authority. The feasibility that each attack can take place will be calculated and compared to each other for each scenario [16]. Three scenarios are proposed: traditional central authority, blockchain technology and FlexiChain technology for the listed attacks below:

1. Attack-1: Data Corruption: For this attack, the digital assets exchanged among participants will be corrupted.
2. Attack-2 Implant Incorrect Data: For this attack, malicious activity by implanting incorrect data takes place while transacting data.
3. Attack-3: Central Authority Full Malicious Control: For this attack, maliciously expose the authority database.

#### 5.2.1. Traditional Central Paradigm

The probability of the attacks to occur in the central paradigm (P(TC)) is given by:

(1) P(TC)=P(A)+P(B)+P(C)

P(A) represents attack-1 and can be performed by a successful attack over all edge nodes and represented as ακ. The probability can be calculated by

(2) $$P\left(A\right)=\frac{1}{4}\prod_{\kappa =1}^{n}\alpha \kappa$$

**P(B1)** represents attack-2 and can be performed by a successful attack over the transmission channels between edge nodes and central node represented as βκ. The probability can be calculated by

(3) $$P\left(B1\right)=\frac{1}{4}\prod_{\kappa =1}^{n}\beta \kappa$$

The corresponding transmission channels are also considered in the formula which can be calculated similar to **P(B1)** and calculated by **P(B2)**.

(4) $$P\left(B2\right)=\frac{1}{4}\prod_{\kappa =1}^{n}\beta \kappa$$

**P(C)** represents attack-3 and can be performed by a successful attack over a central node represented as ρ. The probability can be calculated by

(5) $$P\left(C\right)=\frac{1}{4}\omega$$

From Equations (1)–(4), we obtain the total probability:

(6) $$P\left(TC\right)=\frac{1}{4}\prod_{\kappa =1}^{n}\alpha \kappa +\frac{1}{4}\prod_{\kappa =1}^{n}\beta \kappa +\frac{1}{4}\prod_{\kappa =1}^{n}\beta \kappa +\frac{1}{4}\omega$$

**Figure 10:**
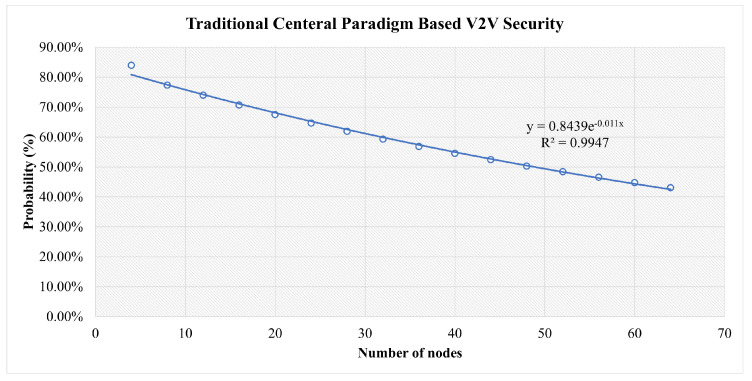
Results for All Three Categories of Attacks in the Traditional Central-Based V2V Scenario.

This is shown as a function of the number of nodes in Figure 10 along with the parameters of an exponential fit of the data.

#### 5.2.2. Blockchain

The probability of the attacks to occur in the blockchain paradigm is

(7) P(BC)=P(A)+P(B)+P(C)

P(A) represents attack-1 and can be performed by a successful attack over all nodes ακ and acquiring nodes’ credentials represented as θκ. The probability can be calculated by

(8) $$P\left(A\right)=\frac{1}{4}\prod_{\kappa =1}^{n}\alpha \kappa \times \frac{1}{4}\prod_{\kappa =1}^{n}\theta \kappa$$

**P(B1)** represents attack-2 and can be performed by a successful attack over nodes and acquiring nodes’ credentials represented as θκ. There is $\frac{n\times (n-1)}{2}=a$, which is the number of transmissions channels represented as βκ, which can be created by pairs of nodes for *n* nodes [16]. The probability can be calculated by

(9) $$P\left(B1\right)=\frac{1}{4}\prod_{\kappa =1}^{a}\beta \kappa \times \frac{1}{4}\prod_{\kappa =1}^{n}\theta \kappa$$

The corresponding transmission channels are also considered in the formula which can be calculated similar to **P(B1)** and denoted as **P(B2)**.

(10) $$P\left(B2\right)=\frac{1}{4}\prod_{\kappa =1}^{a}\beta \kappa \times \frac{1}{4}\prod_{\kappa =1}^{n}\theta \kappa$$

**P(C)** represents attack-3 and can be performed by a successful attack over all edge nodes ρκ and acquiring nodes’ credentials represented as θκ. There are $\frac{n}{2}=v$, which is the number of mining nodes or validators (edges) that an attacker should control to compromise the ledger. The probability can be calculated by

(11) $$P\left(C\right)=\frac{1}{4}\prod_{\kappa =1}^{v}\rho \kappa \times \frac{1}{4}\prod_{\kappa =1}^{n}\theta \kappa$$

(12) $$\begin{array}{c} P\left(BC\right)=\frac{1}{4}\prod_{\kappa =1}^{n}\alpha \kappa \times \frac{1}{4}\prod_{\kappa =1}^{n}\theta \kappa \\ +\frac{1}{4}\prod_{\kappa =1}^{a}\beta \kappa \times \frac{1}{4}\prod_{\kappa =1}^{n}\theta \kappa \\ +\frac{1}{4}\prod_{\kappa =1}^{a}\beta \kappa \times \frac{1}{4}\prod_{\kappa =1}^{n}\theta \kappa \\ +\frac{1}{4}\prod_{\kappa =1}^{v}\rho \kappa \times \frac{1}{4}\prod_{\kappa =1}^{n}\theta \kappa \end{array}$$

**Figure 11:**
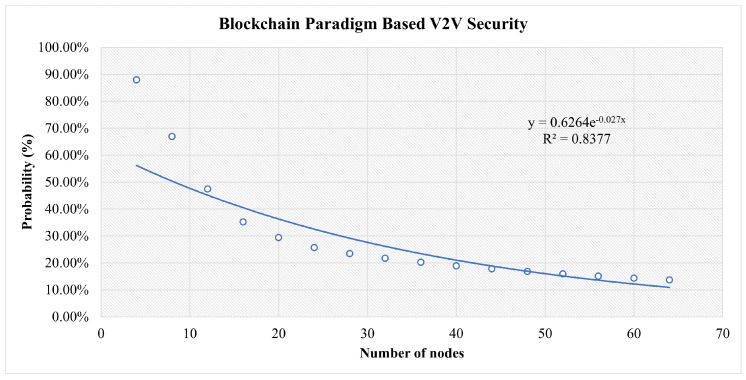
Results for Three Categories of Attacks in the Blockchain-Based Scenario.

This is shown as a function of the number of nodes in Figure 11 along with the parameters of an exponential fit of the data.

#### 5.2.3. FlexiChain

The probability of the attacks to occurs in the blockchain paradigm is

(13) P(FC)=P(A)+P(B)+P(C)

P(A) represents attack-1 and can be performed by a successful attack over all nodes ακ, acquiring nodes’ credentials represented as θκ, acquiring trusted attached hardware credentials, and Unique Identification (UID). The probability can be calculated by

(14) $$P\left(A\right)=\frac{1}{4}\prod_{\kappa =1}^{n}\alpha \kappa \times \frac{1}{4}\prod_{\kappa =1}^{n}\theta \kappa \times \frac{1}{4}\prod_{\kappa =1}^{n}\phi \kappa \times \frac{1}{4}\prod_{\kappa =1}^{n}\Phi \kappa$$

**P(B1)** represents attack-2 and can be performed by a successful attack over all nodes ακ and acquiring nodes’ credentials represented as θκ. There are $\frac{n\times (n-1)}{2}=a$, which is the number of transmissions channels that can be created by pairs of nodes for n number of nodes [16]. The probability can be calculated by

(15) $$P\left(B1\right)=\frac{1}{4}\prod_{\kappa =1}^{a}\beta \kappa \times \frac{1}{4}\prod_{\kappa =1}^{n}\theta \kappa \times \frac{1}{4}\prod_{\kappa =1}^{n}\phi \kappa \times \frac{1}{4}\prod_{\kappa =1}^{n}\Phi \kappa$$

The corresponding transmission channels are also considered in the formula which can be calculated similar to **P(B1)** and denoted as **P(B2)**.

(16) $$P\left(B2\right)=\frac{1}{4}\prod_{\kappa =1}^{a}\beta \kappa \times \frac{1}{4}\prod_{\kappa =1}^{n}\theta \kappa \times \frac{1}{4}\prod_{\kappa =1}^{n}\phi \kappa \times \frac{1}{4}\prod_{\kappa =1}^{n}\Phi \kappa$$

**P(C)** represents attack-3 and can be performed by a successful attack over all nodes ρκ and acquiring nodes’ credentials represented as θκ. There are $\frac{n}{2}=v$, which is the number of mining nodes or validators (edges) that an attacker should control to compromise the ledger. The probability can be calculated by

(17) $$P\left(C\right)=\frac{1}{4}\prod_{\kappa =1}^{v}\rho \kappa \times \frac{1}{4}\prod_{\kappa =1}^{n}\theta \kappa \times \frac{1}{4}\prod_{\kappa =1}^{n}\phi \kappa \times \frac{1}{4}\prod_{\kappa =1}^{n}\Phi \kappa$$

(18) $$\begin{array}{c} P\left(FC\right)=\frac{1}{4}\prod_{\kappa =1}^{n}\alpha \kappa \times \frac{1}{4}\prod_{\kappa =1}^{n}\theta \kappa \times \frac{1}{4}\prod_{\kappa =1}^{n}\phi \kappa \times \frac{1}{4}\prod_{\kappa =1}^{n}\Phi \kappa \\ +\frac{1}{4}\prod_{\kappa =1}^{a}\beta \kappa \times \frac{1}{4}\prod_{\kappa =1}^{n}\theta \kappa \times \frac{1}{4}\prod_{\kappa =1}^{n}\phi \kappa \times \frac{1}{4}\prod_{\kappa =1}^{n}\Phi \kappa \\ +\frac{1}{4}\prod_{\kappa =1}^{a}\beta \kappa \times \frac{1}{4}\prod_{\kappa =1}^{n}\theta \kappa \times \frac{1}{4}\prod_{\kappa =1}^{n}\phi \kappa \times \frac{1}{4}\prod_{\kappa =1}^{n}\Phi \kappa \\ +\frac{1}{4}\prod_{\kappa =1}^{v}\rho \kappa \times \frac{1}{4}\prod_{\kappa =1}^{n}\theta \kappa \times \frac{1}{4}\prod_{\kappa =1}^{n}\phi \kappa \times \frac{1}{4}\prod_{\kappa =1}^{n}\Phi \kappa \end{array}$$

**Figure 12:**
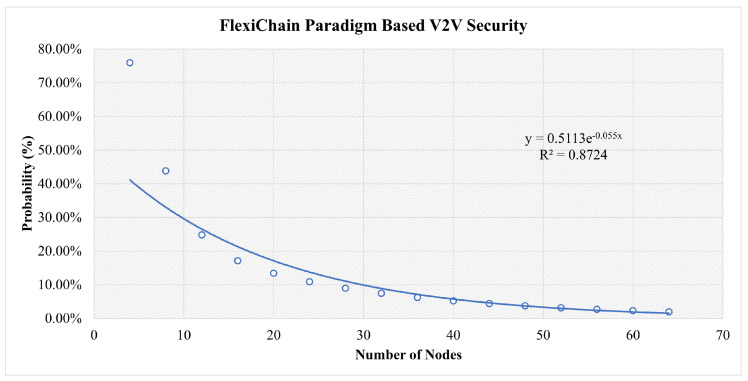
Results for Three Categories of Attacks in the FlexiChain-Based Scenario.

This is shown as a function of the number of nodes in Figure 12 along with the parameters of an exponential fit of the data.

### 5.3. Comparative Analysis of FlexiChain 3.0

A comparative analysis of the three types of DLT examined in this work is given in Table 6.

A simulation has been performed over Equations (6), (12), and (18). For factors α, θ, ϕ, and Φ, values in the (0.9–1) range are assumed and will be assigned for each based on the difficulty of an attack [16]. For ω, it is assumed a value of (0–0.1) will be assigned [16]. The number of nodes chosen is 4 to 64.

It is seen from Equations (6), (12) and (18) that FlexiChain has more security layers, which would make any malicious attack very expensive. In addition, another factor is the number of nodes that play a major role in the feasibility of an attack. The more nodes required, the less vulnerable the network, Table 7. The quality of the exponential regression (in terms of the correlation coefficient $R^{2}$) is tabulated in Table 8.

**Figure 13:**
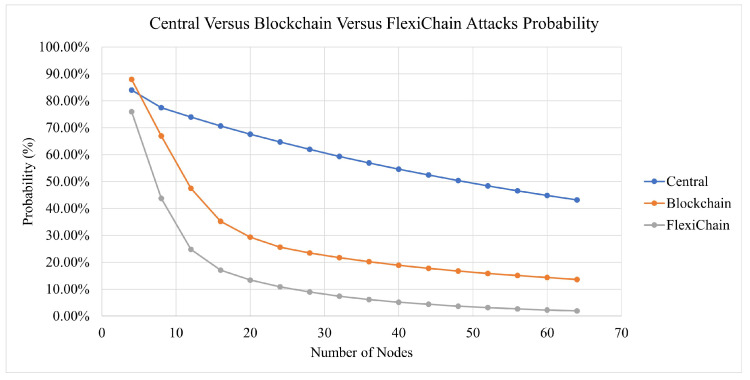
Chart Comparison between Central, Blockchain and FlexiChain.

In Figure 13, in the early stages, all scenarios are at high risk. However, the more nodes that join the network, the more stable it becomes. The traditional central scenario needs a huge number of nodes to reach a stable security and for our simulation for the highest number of nodes used, it reaches 43% security vulnerability to attacks. For Blockchain-based V2V, the same factors play a role. However, the decentralization and distributed authority have reduced the risk to less than 14% at a secure stage. In FlexiChain, based on the multiple factors shown in Table 6, the security risk level has been further reduced from an earlier stage due to fairness of authority distribution, complete decentralization, and the extra security layers.

However, up to some stage, the curve will run close to zero in blockchain and FlexiChain, as shown in Figure 10, Figure 11 and Figure 12. All curves follow an exponential decay that declines toward zero. However, in blockchain and FlexChain, it is obvious that the dependency is lower, since the network has more security factors, as shown in Table 6. In addition, the threat level decreases faster in FlexiChain than in blockchain due to there being more security levels. A comparative perspective with previous works is given in Table 9 and Table 10.

## 6. Conclusions and Future Directions

ITS, the IoV, and VANETs can be transformed by DLT. A DLT’s decentralization, transparency, security, and immutability can help stakeholders address data sharing, trust management, and privacy issues in these networked systems.

DLT can create secure data-sharing platforms, efficient payment systems, and decentralized marketplaces for vehicle digital assets and services in ITS, IoV, and VANETs. Smart contracts automate processes, improving stakeholder transactions.

Despite its promise, DLT implementation in ITS, IoV, and VANETs must address scalability, latency, energy efficiency, and privacy issues. DLT’s full potential in establishing intelligent, safe, and sustainable transportation systems in smart cities depends on further research and development in these areas and the deployment of proper consensus algorithms and blockchain platforms.

FlexiChain Technology 3.0 has been proposed as an ITS platform that could provide safe and secure operation and a scalable architecture that could match the expected operation volumes in the IoV. FlexiChain 3.0 achieved 2.3 trx/s, which is not an optimal target but adequate to serve IoV. However, these results provide motivation to optimize the implementation and reduce latency by using better mechanisms to elect a block location. Security analysis has been introduced to show that the security measures used in FlexiChain were a match to the ones used in the blockchain. The difference is that FlexiChain is BlockDAG, and its highest security is achieved early stages.

The integration between DLTs and AI will complete the missing pieces of both technologies. AI is a future target to integrate FlexiChain with Deep Reinforcement Learning models for better authority distribution and autonomous authentication.

The next mode of transportation, the Decentralized Intelligent Transportation System (DITS), is still in its infancy. Research into self-driving cars has begun at several universities and businesses, indicating that the suggested work will be needed in the not too distant future. Vehicle-to-vehicle communication is expected to speed up the development of autonomous vehicles.

## Figures and Tables

**Table 1:** Comparative Perspective between FlexiChain Versions.

Features/Version	FlexiChain 1.0 [30]	FlexiChain 2.0 [31]	FlexiChain 3.0 (Current)
Linked Lists	Genesis Blockchain	Layer0 Ledger	Independent NodeChain
	(independent ledger)	Independent NodeChain	Layer0 Ledger
	DAG-Based Blocks	DAG-Based Blockchains	DAG-Based BlockX
Registration	Pre-Installed or	Trusted Security Hardware	Entry Key Pre-Enrollment
	Equipped Manufacturer Trusted Modules	NodeChain (Chain of Narration)	Pre-Launched NodeChain Manufacturer Trust
Authentication	ASID Proof of Rapid Authentication	NodeChain Proof of Rapid Authentication	NodeChain Proof of Rapid Authentication
Type of Validation	Authentication (Minerless)	Authentication (Minerless)	Authentication (Full Nodes)
Validators	All Virtual Nodes	All Qualified Nodes	Full Nodes
Security	Digital Signature	Digital Signature,	Digital Signature
	Hash Function	Hash Function	Hash Function
	Secure File (ASID) and TPM	Tokenized UID and Hardware Security	Tokenized UID from Independent Offline NodeChain
Design Purpose	IoT/CPS Applications	IoT/CPS Applications	Intelligent Transportation

**Table 2:** Comparison of Consensus Algorithms and DLTs.

#	Algorithm/Technology	Pros	Cons
1.	Proof of Work (PoW) [49]	High security, Decentralization	Energy inefficiency, Scalability issues
2.	Proof of Stake (PoS) [50,51]	Energy efficiency, Faster transactions	Centralization risk, Security concerns
3.	Delegated Proof of Stake (DPoS) [51]	High scalability, Energy efficiency	Centralization risk
4.	Practical Byzantine Fault Tolerance (PBFT) [52]	Fast transactions, High fault tolerance	Limited scalability, Centralization risk
5.	Proof of Authority (PoA) [53]	Fast transactions, Energy efficiency	Centralization risk
6.	Tangle (DAG-based) [54]	Scalability, No transaction fees, Energy efficiency	Vulnerable to spam attacks, Lower security guarantees
7.	Hashgraph [55]	Fast transactions, Fairness, Asynchronous Byzantine Fault Tolerance	Centralization risk, Licensing and patent issues

**Table 3:** A Comparative Perspective of DLT-Based Previous Works.

Decentralized Applications (Dapps)	DLT Type	Consensus Algorithm	Resources Requirements	Contributions
Kang et al., 2019 [58]	Consortium Blockchain	Proof of Work (PoW)	High	Blockchain-based data management using smart contract
Javaid et al., 2019 [59]	Public Blockchain	Proof of Work	High in RSU, Low in IVs	Blockchain-based trust management and data exchange using smart contract
Chen et al., 2019 [61]	Consortium Blockchain	Proof of Work (PoW)	High in Edge Layer, Low in Vehicles Layer	Vehicle data trading blockchain based
Zichichi et al., 2020 [65]	Permissionless DLT	IOTA Proof of Work	Medium/Low	Data Management and services decentralized framework using IOTA as a distributed ledger, Ethereum Virtual Machine smart contracts and distributed database such IPFS
Maffiola et al., 2022 [66]	Consortium Blockchain	Proof of Stake (PoS) and PBFT	Medium	Blockchain data collection framework

**Table 4:** Setup Components.

Number of Nodes	Size of Block	Up Time (s)
64	32 × 1024 bytes	1800

**Table 5:** FlexiChain Technology Number of Transactions/Second.

Nodes	Total Number of Blocks	Trxs/Second (Average)
64	4158	2.3

**Table 6:** Comparison Between Central Versus Blockchain Versus FlexiChain.

Attack Type	Central Based V2V	Blockchain Based V2V	FlexiChain Based V2V
Attack-1 (A)	Equation (2): 1 security factor	Equation (8): 2 security factor	Equation (14): 4 security factors
Attack-2 (B)	Equations (3) & (4): transmission channel between server and edge nodes	Equations (9) & (10): transmission channels combinations between 2 nodes. Creates more channels to be compromised which increased cost for adversary	Equations (15) & (16): transmission channel combinations between 2 nodes. Creates more channels to be compromised which increase cost for adversary
Attack-3 (C)	Equation (5): 1 authority capacity	Equation (11): Validators = Authority Capacity	Equation (17): All = authority capacity

**Table 7:** Comparative Analysis between Central Versus Blockchain Versus FlexiChain: This table shows the probabilities acquired from our security analysis for three categories of attacks for three scenarios.

Number of Nodes	Central (%)	Blockchain (%)	FlexiChain (%)
4	84%	87%	76%
24	65%	26%	11%
44	52%	18%	4%
64	43%	14%	2%

**Table 8:** Quality of Regression of Attack Success Probability to P=aexpbx.

Figure	*a*	*b*	$R^{2}$
Figure 10	0.84	0.011	0.99
Figure 11	0.62	0.027	0.837
Figure 12	0.51	0.055	0.872

**Table 9:** A Perspective Comparative of the Proposed Consensus Algorithm with Related Works.

Consensus Algorithm	Ledger	Tolerance	DLT Type	Trust Type
Proof of Work (PoW) [67]	Full	<25%	Public/Permissionless	No Trust
Proof of Stake (PoS) [67]	Full	<51%	Public/Permissionless	No Trust
Byzantine Fault Tolerance (BFT) [68]	Full	<33.3%	Private/Permissioned	Trust
Federated BFT (FBFT) [69]	Full	<33.3%	Private/Permissioned	Trust
Practical BFT (PBFT) [69]	Full	<33.3%	Private/Permissioned	Trust
Proof of Authority (PoA) [70,71]	Full	<51%	Public/Permissioned	Trust
NPoRa Figure 7 (Current)	Portion/Full	<100%	Public/Permissioned	Manufacturing Trust

**Table 10:** Comparative Analysis between Current Consensus Algorithm with Related Works.

Attributes	PoW	PoS	DPoS	LPoS	PoI	PoA	PoET	PoB	PoC	BFT	PBFT	Tangle	NPoRa
Hardware Requirements	H	M	M	M	M	M	M	M-H	M	M	M	L-M	L-M
Pre-Trust Level	L	M	M	M	M	H	M	M	M	H	H	M	L-M
Tolerance Level	M	M	M	M	M	H	M	M	M	H	H	L-M	H
Overhead Computation	H	M	L	M	M	L	L	M	M	M	M	L	L
Centralization Level	M	M	M	M	M	H	M	M	M	H	H	M-H	L
Scalability Level	M	M	M	M	M	H	M	M	M	H	H	H	H
Latency Level	M	M	M	M	M	H	M	M	M	H	H	L	L-M
Cost Level	M	M	M	M	M	H	M	M	M	H	H	M	L
Security Level	M	M	M	M	M	H	M	M	M	H	H	M	H
ITS Compatibility	L	L	L	L	L	L-M	L	L	L	L-M	M-H	H	H

Definitions: H: High; M: Moderate; L: Low. Definitions: Pre-hardware requirements: The necessary computing resources, components, and infrastructure that a node must possess to participate effectively in the network. Pre-trust level: The inherent trust assumptions or level of trust required for each consensus mechanism to function properly. Tolerance level: The resilience and adaptability of a consensus mechanism under various adversarial conditions or system failures. Overhead computation: The additional processing effort or resources required by nodes to participate in the consensus process and maintain the network. Centralization level: The degree to which control, decision-making power, and resources are concentrated within a network. Scalability level: The ability of a network to handle an increasing number of transactions and participants while maintaining acceptable performance levels. Latency level: The amount of time it takes for a transaction to be processed, confirmed, and added to the ledger. Cost level: The various expenses associated with operating, maintaining, and participating in a network. Security level: The ability of a network to protect itself from various threats, such as cyber-attacks, fraud, and manipulation.

## Data Availability

The data presented in this study are available on request from the corresponding author. The data are not publicly available due to ongoing research and analysis.
